# Perseverance of Effort and Consistency of Interest: A Longitudinal Perspective

**DOI:** 10.3389/fpsyg.2021.743414

**Published:** 2021-08-30

**Authors:** Rouhua Wang, Majid Elahi Shirvan, Tahereh Taherian

**Affiliations:** ^1^School of Foreign Languages, Changchun Institute of Technology, Changchun, China; ^2^Department of Foreign Languages, University of Bojnord, Bojnord, Iran; ^3^Department of English Language and Literature, Yazd University, Yazd, Iran

**Keywords:** L2 grit, perseverance of effort, consistency of interest, factor of curves modeling, latent growth curve modeling

## Abstract

The present research, enlightened by the dynamic approach to language learning, aimed to trace the co-development of English as a foreign language (EFL) learners’ perseverance of effort (PE) and consistency of interest (CI), the two subdomains of grit, over a course time. To this aim, a factor of curves model (FCM) was employed to trace the covariance of the subdomains of L2 grit longitudinally over time; to explore how changes in PE and CI are affected by their initial states; and to what extent the variations in PE and CI are explained by the underlying global factor of L2 grit. For data collection, the L2 grit scale was used with 1,384 adult EFL learners in four measurement occasions. The data were analyzed in Mplus with FCM in three steps. The results showed that firstly, in analyzing the direction of change in PE and CI, a higher mean was estimated in the growth (the slope) than the intercept. Evidence was also found for inter-individual variation. Secondly, the covariance between the intercept and slope of each subdomain was explored and for both the results revealed a negative covariance of the slope and intercept. Thirdly, the covariance of the intercepts and the slopes of the two subdomains was positive and significant. Fourthly, the variance of the intercept and slope of each subdomain proved to be explained to a large extent by the underlying global factor of L2 grit. These findings were discussed in the light of the potential variables associated with L2 grit and its related literature.

## Introduction

A non-cognitive skill and a personality construct recently introduced and shown to be promising in life success as well as academic success is grit (McCain, 2017). It has been defined along two dimensions of perseverance of effort (PE) and consistency of interest (CI; Duckworth et al., 2007). Both dimensions of grit contribute significantly to success as perseverance of effort facilitates the achievement of mastery in spite of failure, and consistency of interest is a key to deliberate practice to gain mastery (Credé et al., 2016). The distinction between the two dimensions of grit was made in the grit scale with 12 self-rating items developed by Duckworth et al. (2007) and a short version (known as the grit scale-S) with eight items developed by Duckworth and Quinn (2009). Strong claims were made about grit by Duckworth (2013) to introduce it as even a better predictor of success than cognitive ability (Credé et al., 2016). Advocating the importance of grit relates also to the works of research on the development of expertise with an increasing attention to continuous deliberative practice (Ericsson et al., 1993; Krampe and Ericcson, 1996). Besides, there are several theoretically sound moderators of the relationship between grit and academic performance that show the relation is at best context-dependent. In other words, how grit relates to academic outcome is moderated by the nature of the performance domain. If the task is demanding but well-defined, then high grit can be truly useful. Actually, high levels of continuous effort and deliberative practice are needed for success and how performance is to be rewarded is relatively clear (Credé et al., 2016).

In second language acquisition (SLA) domain, grit has been largely under-researched and the earliest study of the construct was by Lake (2013) using the general grit scale. Why this construct has been scarcely investigated in SLA is partly due to the measurement inadequacy of the construct (Khajavi et al., 2020). The domain-specific L2 grit scale has been recently developed by Teimouri et al. (2020) with nine items (five measuring PE and four measuring CI) on a five-point Likert scale. Thus, the existing body of research on grit in SLA is still in its infancy. Yet, the few studies conducted so far will be reviewed here.

## Literature Review

In the SLA domain, the early works of research used the general grit scale to measure language learners’ grit. To begin with, Lake (2013) investigated the correlation between grit and a number of positive psychological variables. He surveyed 800 female Japanese university students who learned English language *via* a modified edition of Duckworth et al.’s (2007) general scale and found that grittier students have a stronger interest in spending time and putting efforts in L2 learning. In another investigation, Changlek and Palanukulwong (2015) surveyed 183 Thai students to explore gritty students’ motivational characteristics. They found that high achievers were grittier and grit was positively correlated with students’ motivation and negatively with anxiety.

A modified version of the general grit scale was also used by Kramer et al. (2017) to test the predictive validity of grit in a sample of foreign language learners in Japan *via* a Rasch analysis. Correlation analyses showed a moderate association between L2 learners’ vocabulary scores and grit. These researchers also found a minor correlation between L2 learners’ grit and the amount of words read throughout a long reading task. The general grit scale was also used by Robins (2019) to explore the effect of grit on retention and academic success of online English as a second language (ESL) learners in the United States. The overall grit score did not show to be a significant predictor of retention. Yet, it was slightly but significantly correlated with L2 learners’ average score (GPA).

In a similar vein, Wei et al. (2019) found a positive but low correlation between grit and English language scores of 832 secondary school students in China. Yamashita (2018) estimated no correlation between 78 Japanese learners’ grit and their GPA. Interestingly, the PE dimension of grit showed to be negatively correlated with GPA for a sub-sample of the population. It is noteworthy that these studies all used the general grit scales developed by Duckworth and colleagues (Duckworth et al., 2007; Duckworth and Quinn, 2009) and assessed learners’ grit globally and not locally and in relation to the context of L2 learning.

These inconclusive results attested to the fact that an L2-specific grit scale was needed to act as a more accurate measurement of success in an L2 environment (Sudina et al., 2020). Consequently, Teimouri et al. (2020) developed the L2 grit scale to measure the domain-specific grit in SLA. Their findings based on a sample of 191 EFL learners, showed higher correlations between the L2 grit scale and L2 learners’ outcome than the Grit-O, as evidenced by sizeable attenuation-corrected correlations for the L2 grit.

Also, Sudina et al. (2020) investigated the factor structure of the L2 grit scale designed by Teimouri et al. (2020) through exploratory factor analysis, confirmatory factor analysis (CFA), and multiple regression analysis. They showed that the L2 grit scale enjoyed a sufficient internal consistency. A principal components analysis indicated a two-component structure and substantiated the construct validity. A one-tailed Pearson’s test for positive correlation between domain-general Grit-S and L2 grit scale scores established the concurrent validity of the new measure. Finally, the L2 grit scale showed a higher predictive validity, accounting for about 21% of the variance of L2 teacher retention-related scores in comparison with the Grit-S. Furthermore, replicating Teimouri et al. (2020)’s research of L2 grit in the Chinese EFL context, Wei et al. (2020) confirmed the factorial structure of L2 grit scale in this context; more specifically, one of the highlights of this study was that these researchers proposed a more refined data analysis method based on hierarchical regression.

What is common among the existing literature is that the L2 grit works of research are not only limited in size, but also they scarcely address the dynamic nature of the construct. It appears that grit has been recently translated into the domain-specific sphere, yet it has not entered the dynamic phase of research.

### L2 Grit Dynamics

Besides the need for further in-depth domain-specific investigations of grit in language learning, attention to the developmental and growing nature of the construct is inevitable. Admittedly, within the last decade, the bulk of grit-related studies have been criticized for neglecting the situational and context-specific nature of the construct (Christopoulou et al., 2018). Very recently, putting emphasis on the dynamic nature of L2 grit, having examined L2 grit longitudinally, Alamer (2021) found that initial level of L2 grit could not explain later variance of achievement in L2 vocabulary. He also found that sustained endorsement of grit could predict the increase in vocabulary achievement, denoting the temporal change of L2 grit.

It should be noted that addressing the construct from the perspective of complex dynamic systems theory (CDST) approach helps to capture the nuances of changes in grit and its dimensions, PE and CI. From a CDST perspective, more aspects of the task, person, and environment are measured in combination to obtain a more representative measurement of a trait (Darrah and Bartlett, 1995). In order to measure the longitudinal nature of L2 grit as it is actually experienced during an L2 course and to trace the co-development of its two dimensions (i.e., PE and CI), conventional statistical methods (e.g., regression analysis, mean comparisons, and repeated-measures analysis of variance) are deemed inadequate (Wickrama et al., 2016). They fail to account for measurement errors, measurement invariance, inter-individual differences, co-development of sub-constructs and sensitivity to the initial status of the construct, and the rate of change in the construct and sub-constructs. Innovative statistical procedures within structural equation modeling (SEM), such as parallel process modeling (PPM) and factor of curve modeling (FCM), however, are capable of providing a more realistic image of the construct and its dimensions as they are measured over time in an educational course (Wickrama et al., 2016). These innovative statistical techniques, though still scarcely used to measure personality constructs in SLA, is involved in language learning, manage to embrace the dynamicity of personality constructs as they naturally develop in the ecology of classroom (Hiver and Al-Hoorie, 2019). Here, more specifically, the FCM approach will be introduced.

### The FCM Approach to L2 Grit Dynamics

The FCM approach is most appropriate when the aim is to investigate the co-development of time-varying attributes because it allows for the examination of both trajectories of specific subdomains and trajectories of the global, second-order, domain during stages or phases of rapid development (Wickrama et al., 2016). Contrary to growth curves models that use composite measures and estimate fewer parameters, FCM takes into account the differential contribution of the primary growth factors (here, the level and slope of PE and CI) to the second-order factors of the model (the initial state and the slope of change in grit). It is called a factor-of-curves model because it first estimates growth curves and then defines factors of these curves (Duncan and Duncan, 1996).

Higher-order modeling frameworks, such as FCM, allow different subdomains of L2 grit (CI and PE) to fluctuate differently over time by forming primary growth factors that are specific to the dynamicity of each subdomain. The primary growth factors of these subdomains are used as multiple indicators of higher-order factors of L2 grit. The significant covariance among primary growth factors can be represented by the underlying secondary growth factors, forming a second-order growth curve model known as an FCM. In this extension of a PPM, the covariances among first-order growth factors are hypothesized to be explained by the second-order growth factors (Wickrama et al., 2016). For example, the covariance between the initial level of PE and the initial level of CI can define a second-order initial level factor, while the covariance between the slope of PE and the slope of CI can define a second-order slope factor. Together, these second-order growth factors form a second-order growth curve representing a global domain of L2 grit comprised of two subdomains (PE and CI). With these explanations in mind, in this study, we were interested in investigating whether a second-order factor structure of the global domain of L2 describes the associations among primary growth factors of CI and PE.

For a successful implementation of the FCM approach, evidence of the desired longitudinal pattern of correlations among within-subdomain repeated measures and significant correlations among primary growth factors for the formation of second-order growth factors is needed. We also need evidence for high correlations among time-specific measures of the target subdomains and the necessary longitudinal pattern of correlations among confirmatory latent factors. These are also tested and confirmed in the present research.

## Research Questions

In this study, we aimed to trace the co-development of L2 learners’ CI and PE (two subdomains of L2 grit), to measure the relative effect of the initial state of each subdomain on the development of both over time, to test the significance of changes in PE and CI, and to assess the predictive power of the intercept and slope of L2 grit on the intercept and slope of changes in PE and CI, the FCM approach. The research questions are as follows:

RQ1: To what extent and in what direction do EFL learners’ PE and CI change over time?
RQ2: To what extent are changes in PE and CI associated with their initial levels?
RQ3: To what extent are changes in PE and CI related over time?
RQ4: To what extent does the global factor of L2 grit account for the variances of first-order growth parameters of PE and CI?

The research questions attempt to reflect the dynamic nature of the construct as ordinarily expected from a longitudinal perspective and as recently suggested to be explored through latent growth modeling (LGM; Hiver and Al-Hoorie, 2019). Yet, the distinctive feature of the present study is that it goes beyond tracing L2 grit as a univariate construct (as measured in LGM). Rather, the FCM procedure employed here provides for checking the growth changes of the two subdomains of grit through time influenced by the initial level of each and the global domain of L2 grit. Thus, FCM helps to more delicately reveal the dynamicity of the construct and its subdomains, in a way scarcely experienced before in SLA domain.

## Methodology

The present research used the FCM approach to explore EFL learners’ grit along the two dimensions of CI and PE. There were certain steps to follow, as suggested by Wickrama et al. (2016), as summarized here.

Step 1: Investigating the Longitudinal Correlation Patterns Among Repeated Measures of Each Subdomain

The FCM has a measurement model consisting of multiple primary growth curves at the first level. For the successful estimation of primary growth curves, the associations among repeated indicators at adjacent occasions should be consistently higher than associations at non-adjacent measurement occasions (Lorenz et al., 2004).

Step 2: Estimating a Parallel Process Growth Curve Model (PPM)

PPM helps researchers to examine the correlation of primary growth parameters for multiple subdomain trajectories (Duncan et al., 2006). The first step for developing this model is to ensure that each primary growth curve model has been successfully modeled independently (known as checking model fit). Next, it is essential to determine from each of the primary growth models whether there is enough inter-individual variation in the initial levels and slopes. Significant variations in the intercepts and slopes prove that forming a PPM is a possibility. For example, univariate primary latent growth curve models (LGCMs) can be estimated using two or three subdomains. Then, growth parameters (i.e., intercept and slope variance) of each model can be correlated.

Step 3: Estimating a FCM

To gain empirical evidence of the accurate estimation of second-order growth factors for an FCM, the covariances (or correlations) among primary growth factors, which are the indicators of the second-order growth curve, should be examined first. Even if the second-order model explains all of covariations among the primary growth factors, the model fit indices may not improve over those of the corresponding primary growth curve models. Yet, if the model fit indices for the second-order growth model are close to those of the corresponding primary growth models, Duncan and Duncan (1996) argue that an FCM can still be conceived an acceptable model as it is more parsimonious.

There are certain expectations when estimating a FCM. For one, we expect that primary, or first-order, growth factors (e.g., the initial level and slope) of a subdomain describe the developmental change in that specific subdomain over time. For another, we expect that the covariations between growth factors of different subdomains reflect a parallel process involving different subdomains over time. In addition, we expect higher-order factors (second-order or global factors) to accurately reflect the variances and covariances among the primary growth factors. Overall, the suggested steps to conduct an FCM procedure are as: (a) to investigate the correlation among indicators of each subdomain over time to check the possibility of forming primary growth curves for each subdomain, (b) to estimate a parallel process growth curve model (PPM) comprised of primary growth curves for each subdomain with correlations among primary growth factors and measurement errors, and (c) to estimate the FCM (Wickrama et al., 2016).

### Participants and Setting

The population for the aims of this study was EFL/ESL language learners. From this population, 1,384 Chinese English language learners, with Chinese as their first language, were randomly selected from the language institutes of five big cities in China. A total number of 569 participants were female and 815 were male. Based on Oxford Placement Test, their language proficiency ranged between lower-intermediate levels to upper-intermediate level. Their age ranged from 19 to 43years. The data were collected in the autumn 2020 in the context of China.

### Instrumentation

The present study used the L2 grit scale developed by Teimouri et al. (2020) which consists of two related sub-constructs: CI and PE in learning a language. The former addresses changes of students’ interest during L2 learning and the latter measures how persistent learners are in the achievement of their L2 goals. After a series of statistical analyses including item analysis, reliability analysis and principal component analysis, Teimouri et al. (2020) retained 12 items, six measuring consistency of interest (e.g., My interests in learning English changes from year to year) and six measuring perseverance of effort (e.g., I will not give up learning English until I master it). Further analysis showed that the item-total correlations for three items fell below the minimum criteria of 0.40 and were, thus, discarded. The nine remaining items were then used to form the L2 grit scale. They also reported higher reliability for language domain-specific grit than domain-general grit [t(190)=84.34, *p*<0.001, Cohen’s d=0.81]. All the items of the scale were measured using a five-point Likert-type scale ranging from 1 “not at all like me” to 5 “very much like me.” We used Cronbach’s *α* to assess its internal consistency and McDonald’s *ω* (McDonald, 1985, 1999) to check its composite reliability Şimşek and Noyan (2013). Cronbach’s *α* (95% CI) for the CI, PE, and the global L2 grit was 0.90 (0.89–0.92), 0.89 (0.88–0.90), and 0.88 (0.87–0.90), respectively. The McDonald’s *ω* (95% CI) for CI, PE, and the global L2 was 0.91 (0.89–0.93), 0.90 (0.89–0.91), and 0.89(0.88–0.90), respectively. The analyses of invariance measurement (see Kline, 2016) of the L2 grit subscales indicated that the configural model, or the unconstrained model (first model), had a good overall fit [*χ*^2^(df)=63.49 (51), *p*<0.05; CFI/TLI=0.946/0.973; RMSEA=0.037; and SRMR=0.031]. In the configural model, the factor loading of one indicator is set to one for each point of time (PE1~4), and its intercept is set to zero for model identification purposes (for further information see Kline, 2016). Next, we formed the second model by constraining the factor loadings to be equal. We checked the assumption of weak invariance by comparing the second model with the first model. The results showed that the constraints considered in the second model did not significantly reduce the model fit compared to the first model [configural model; Δ*χ*^2^(df)=15.82(6)(for CI); Δ*χ*^2^(df)=17.68 (8) (for PE) *p=* 0.09; ΔCFI=0.004 (for CI) *p=* 0.07; ΔCFI=0.004 (for PE) *p=* 0.08]. Therefore, we met the assumption of weak invariance in both L2 grit subdomains. However, when we compared the second model (the weak invariance model) to the third model (the strong invariance model), imposing constraints to make the manifest variable means equal across time, the constraints imposed on the third model led to a significant reduction of the model fit [significant increase in chi-square; Δ*χ*^2^(8)=37.64 (for CI), Δ*χ*^2^=39.08 (8) (for PE) *p*<0.001]. However, ΔCFI for private FLE was 0.004 and ΔCFI for CI was 0.003 (for PE). This finding showed the existence of partial strong invariance (not strong invariance) for both L2 grit subscales.

### Data Collection

The data collection method was questionnaire-based. The L2 grit scale with nine items developed by Teimouri et al. (2020) was submitted to participants in four measurement occasions with 2week intervals beginning at the outset of the EFL courses (to take into account the initial status factor). This longitudinal data collection helped to measure changes in the target variable through time. It also allowed for estimating the rate of the changes at each phase and accounted for inter-individual differences. Questionnaire completion was done step by step in class in the presence of one of the researchers. The participants were ensured of the confidentiality of the information they provided.

### Data Analysis

All the required statistical analyses were done in Mplus8.4 with a robust maximum likelihood estimator (MLR). The incremental analytical steps suggested by Wickrama et al. (2016) were followed. These included the investigation of correlation patterns among indicators within each subdomain over time to examine the possibility of forming primary growth curves for each subdomain (step one), the estimation of a parallel process growth curve model (PPM) comprised of primary growth curves for each subdomain with correlations among primary growth factors and measurement errors (step two), and incorporation of the secondary growth factors to form an FCM using the primary growth factors from the PPM as indicators (step three). The first two steps referred to the primary latent growth models (i.e., measurement model), and the last step addressed the estimation of unconditional second-order latent models (i.e., FCM).

For the development of a PPM, first, to ensure the successful modeling of each primary growth curve independently, we examined the model fits. Second, from each of the primary growth models, we checked whether there was adequate inter-individual variation in the intercepts and slopes. Significant variations in the intercepts and slopes provided empirical evidence for the possibility of forming a PPM as well as the estimation of the univariate primary LGCMs *via* the two subdomains of L2grit (i.e., PE and CI).

Auto-correlated measurement errors were also incorporated in the current PPM so as to reduce the chances of model misspecification that led to biased model parameter estimates (Wickrama et al., 2016). Since the main goal of the PPM is to explore longitudinal associations across the primary growth factors of PE and CI, we took into account the association between PE and CI at the same point of time to specify the time-specific measurement errors in the PPM. Also, for the empirical evidence of the successful estimation of second-order growth factors for a FCM, we examined the covariances among the primary growth factors of PE and CI.

To test the model fit, we used goodness of fit indices including comparative fit index (CFI), Tucker-Lewis index (TLI), root mean square error of approximation (RMSEA), and standardized root mean square residual (SRMR). The acceptance criteria were CFI and TLI ≥0.90 and ≥0.95, and RMSEA and SRMR ≤0.08 and ≤0.05, pointing to adequate and excellent fit indices, respectively (Hu and Bentler, 1998; Marsh et al., 2004).

## Results

Corresponding to the incremental steps of conducting an FCM procedure, based on which each research question was developed, the results of analysis are presented here in three steps.

### Investigating the Longitudinal Correlation Patterns Among Repeated Measures of Each Subdomain

The successful estimation of primary growth curves relies on the consistency of higher associations of repeated indicators at adjacent occasions than correlations at non-adjacent measurement occasions (Lorenz et al., 2004). As observed in Table 1, correlation matrix indicated that correlation coefficients between two adjacent occasions (t and t+1) for each subdomain were higher than correlations between non-adjacent occasions (PE rs ranged from 0.29 to 0.51; CI rs ranged from 0.34 to 0.57). Moreover, off-diagonal correlations of the same repeated measure over time (in Table 1) showed to be quite different from each other. These correlations show that a significant slope variation might exist for each subdomain trajectory model.

**Table 1:** Correlation matrix between subdomains.

	PE				CI			
	PE1	PE2	PE3	PE4	CI1	CI2	CI3	CI4
PE1	–							
PE2	0.53	–						
PE3	0.38	0.47	–					
PE4	0.32	0.37	0.48	–				
CI1	0.69	0.51	0.45	0.36	–			
CI2	0.50	0.69	0.48	0.32	0.57	–		
CI3	0.57	0.42	0.79	0.41	0.44	0.49	–	
CI4	0.38	0.42	0.47	0.76	0.42	0.35	0.55	–
Mean	3.58	3.87	4.11	4.39	3.23	3.62	3.80	4.11
Variance	0.23	0.27	0.29	0.21	0.29	0.31	0.32	0.34

*PE, perseverance of effort; CI, consistency of interest*.

### Estimating a Parallel Process Growth Curve Model (PPM)

**Figure 1:**
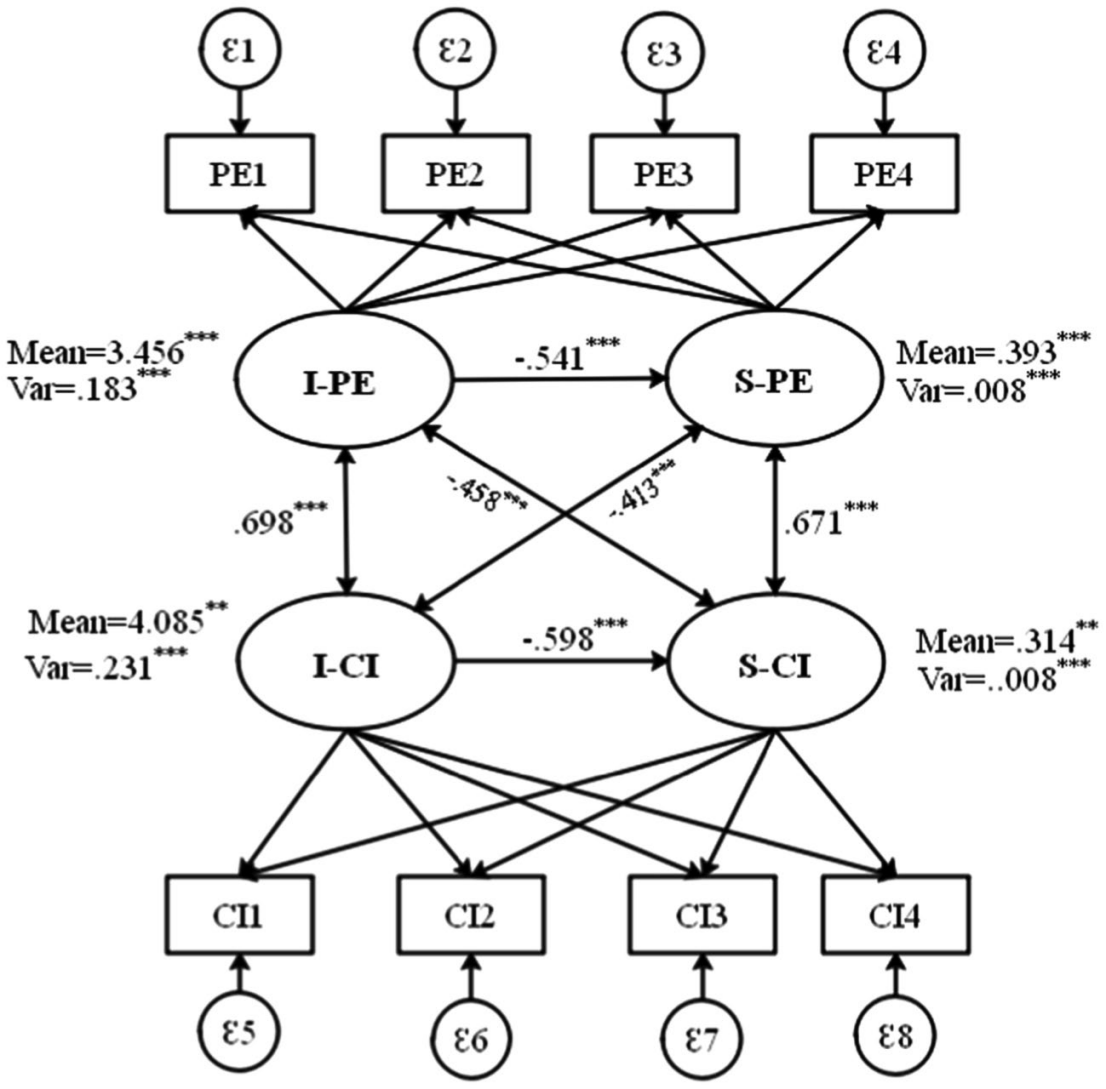
Parallel process model (PPM). I, initial level; S, slope; PE, perseverance of effort; and CI, consistency of interest.

As seen in Figure 1, the intercept and slope variance of each model are correlated. The model results showed that all between-subdomain auto-correlated errors were statistically significant and were within the acceptable bounds, ranging from 0.69 (PE1 with CI1) to 0.76 (PE4 with CI4; see Table 1). Also, the model fit was acceptable [*χ*^2^(df)=198.654(78), *p*<0.001; CFI/TLI=0.945/0.933; RMSEA=0.056; and SRMR=0.051].

The PPM results prove the existence of both between- and within-subdomain auto-correlated error structures. The results of this PPM are illustrated in Table 2. With regard to the first research question regarding the direction and amount of change in PE and CI, the PPM results indicated a statically significant increase over the semester for participants’ PE (M _Intercept_=3.456, SE=0.261, *p* < 0.001; M _Slope_=0.393, SE=0.212, *p* < 0.001) and CI (M _Intercept_=4.085, SE=0.193, *p* < 0.001; M _Slope_=0.314, SE=0.145, *p* < 0.001). Moreover, the intercept variance for PE was 0.183 (*p*<0.001) and CI was 0.231 (*p*<0.001), suggesting that initial PE and CI varied significantly among individuals. In addition, the variances of the slopes of both PE and CI were a significant (0.008, *p*<0.001). This also confirms that there is sufficient inter-individual variation and intra-individual trend within each subdomain, as well as a significant increase over time in both subdomains.

**Table 2:** Results of the parallel process model (PPM).

	Intercept factors		Growth factors		Correlations among growth factors				
	Mean	Variance	Mean	Variance		I-PE	I-CI	S-PE	S-CI
PE	3.456^***^	0.183^***^	0.393^***^	0.008^***^	I-PE	-			
					I-CI	0.694^***^	-		
CI	4.085^***^	0.231^***^	0.314^***^	0.008^***^	S-PE	−0.541^***^	−0.413^***^	-	
					S-CI	−0.458^***^	−0.598^***^	0.671^***^	-

^*^statistically significant.

The covariances among the primary growth factors were also all statistically significant, which is the proof for the existence of a parallel process of growth across the subdomains. It is noteworthy that the correlations (standardized covariances) *among* the intercepts of PE and CI showed to be higher than those *between* the intercepts and slopes of each (see Table 2). In the same vein, correlations *among* the slopes of the two subdomains were higher than the correlations *between* the intercepts and slopes. These relatively strong correlations among the same type of growth parameters across the two subdomains point to the existence of significant global (second-order) growth factors in an FCM.

### Estimating a Factor-of-Curves Model (FCM)

To achieve empirical proof of the successful estimation of second-order growth factors for an FCM, the covariances (or correlations) among primary growth factors of CI and PE, as the indicators of the second-order growth curve, were supposed to be checked first.

**Figure 2:**
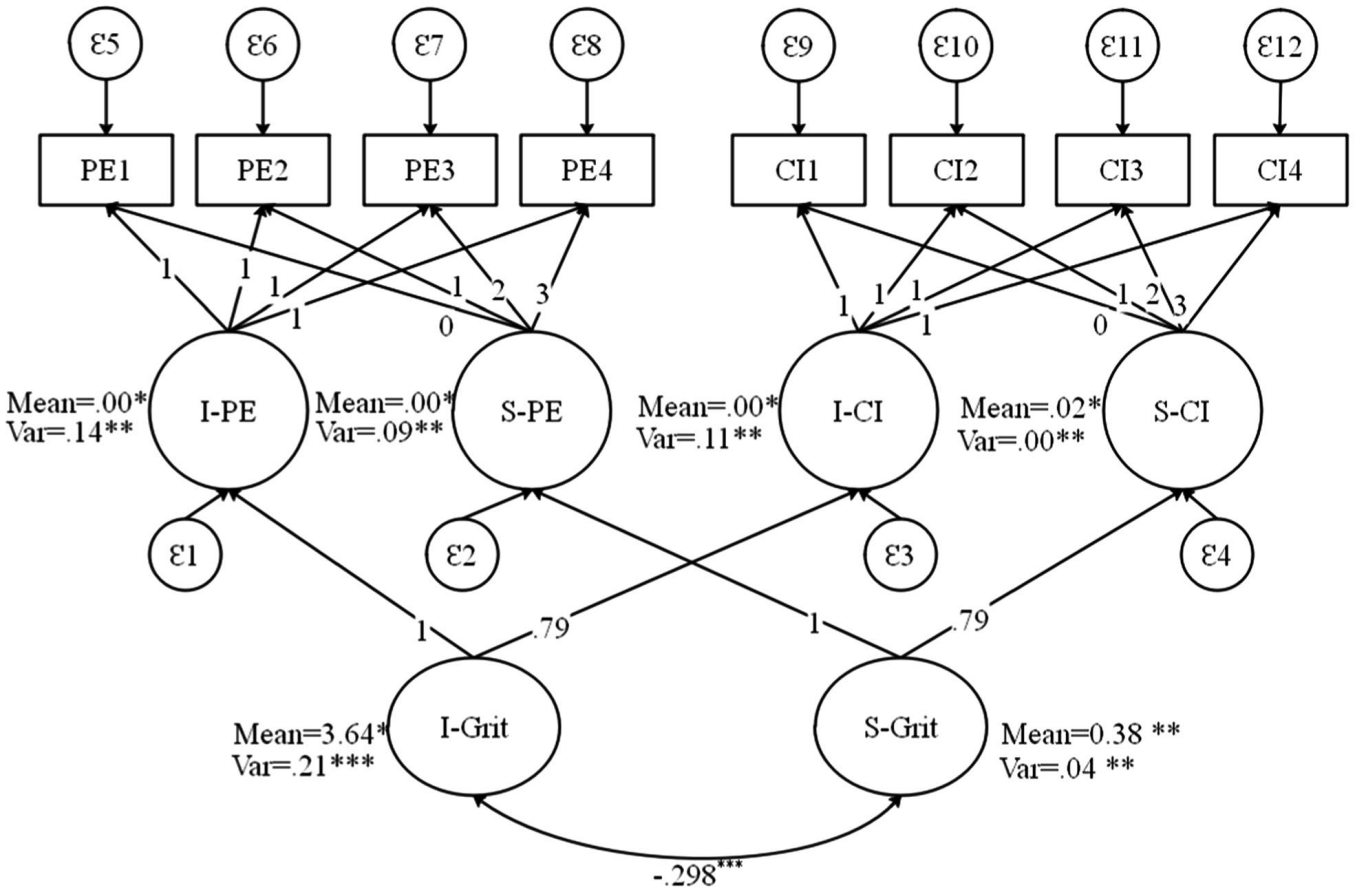
Factor-of-curves model (FCM) of L2 grit. I, intercept; S, slope; PE, perseverance of effort; and CI, consistency of interest.

Regarding the second research question, the association of the initial level of each subdomains with its slope, the PPM results showed moderately high correlations between intercept and slope growth factors within the subdomains of PE and CI (see Table 2). That is, the correlation between the intercept and slope factors for PE was −0.541, *p*<0.001 and that of CI was −0.598, *p*<0.001 (see Figure 2). Given the third research question addressing the association between the changes in PE and CI, as seen in Table 2 in the estimated PPM, the covariances (presented in their standardized form as correlations) between the intercept growth factors were moderately high (*r*=0*.694, p*<0.001). Furthermore, the correlations between the slope factors of PE and CI were also high (*r*=0.671 *p*<0.001; see Figure 2). These findings point to the existence of significant second-order growth factors. These moderately high correlations may show the need to incorporate correlations between the intercept and slope factors of each of the primary growth models in an FCM. In this FCM, we fixed the loadings and means of the PE trajectories to 1 and 0, respectively, so as to estimate the means of the second-order intercept and slope factors using the scale of PE (i.e., marker variable approach).

Here, Table 3 shows that the factor loadings for all of the primary growth factors on the global factors were high and statistically significant, which means that each of the primary growth factors contributed significantly to defining the global factors. The mean and variance of the global intercept were 3.643 and 0.211, respectively. Besides, the mean and variance of the global slope factor were 4.688 and 0.048, respectively. All of these global parameters were statistically significant at the *p*<0.05 level. Regarding the fourth research question, the R-square statistics for each growth factor indicated that the global intercept factor accounted for approximately 84.4 and 61.7% of the variation in the primary intercept factors for PE and CI, respectively. Furthermore, about 83.9 and 61.1% of the variation in the primary slope factors for PE and CI was accounted for by the global L2 grit slope factor. The positive L2 grit slope factor shows an overall decrease in global L2 grit over time. The model results also showed that the growth parameters (i.e., means and variances) of the global intercept and slope factors (I and S) were statistically significant, suggesting that significant inter-individual variation existed for the second-order intercept and slope factor means.

**Table 3:** Parameter estimates for CFM.

	Estimates	S.E.	Est./S.E.	Two-tailed *p*-value
I-Grit by I-PE	1.000	0.000	999.000	999.000
I-Grit by I-CI	0.798	0.058	13.578	0.000
S-Grit by S-PE	1.000	0.000	999.000	999.000
S-Grit by S-CI	0.798	0.058	13.758	0.000
I-PE with S-PE	−0.013	0.002	6.200	0.000
I-CI with S-CI	−0.007	0.003	2.333	0.000
I-Grit with S-Grit	−0.298	0.005	4.500	0.000
**Means**				
I-Grit	3.643	0.038	9.586	0.000
S-Grit	0.388	0.036	13.022	0.001
**Variances**				
I-Grit	0.211	0.034	6.205	0.000
S-Grit	0.048	0.003	1.667	0.000
**Residual variances**				
I-PE	0.032	0.014	2.857	0.000
S-PE	0.000	0.002	0.000	0.001
I-CI	0.014	0.008	1.751	0.032
S-CI	0.064	0.014	4.571	0.041
**R-square**				
I-PE	0.844	0.036	23.301	0.000
S-PE	0.617	0.043	14.209	0.000
I-CI	0.839	0.092	9.793	0.000
S-CI	0.611	0.048	12.979	0.000

Besides, the significant covariance between the intercept and slope of the PE growth model was observed even after controlling for L2 grit growth factors (−0.298, *p*<0.001). These results may prove the existence of unique variance in PE after controlling for secondary L2 grit factors.

## Discussion

Corresponding to the four research questions, there were four major findings in this research to discuss. Firstly, the direction of change in PE and CI was analyzed which showed a higher mean in their growth than their intercept. Besides, we found that the variance of the slope and intercept of each of the subdomains was statistically significant, which attests to the presence of inter-individual variation. A relevant point to raise here is the ergodicity issue concerning language learners. This is embraced in the dynamic systems theory that acknowledges individual differences, emergent behaviors, and contextual sensitivity of human traits or behaviors (Lowie and Verspoor, 2019). Thus, language learners are not supposedly ergodic ensemble and the mean scores of their trait or behavior are not necessarily representative of the reality of the target behavior or trait among individuals. There are cases of individual variation that are covered up in the mean-based estimation of group behaviors or traits. Case studies are, therefore, required to explore the nuances of the trajectory of changes in PE and CI of these individual differences.

Secondly, we explored the covariance between the intercept and slope of each grit subdomain. For both subdomains, the results revealed a negative covariance of the slope and intercept which implies that in each subdomain (PE and CI), learners who started off at a lower level of PE or CI at the beginning of the course experienced a steeper increase in the construct throughout the course than those with a higher initial level of efforts and interest. This can be discussed in a number of ways, yet it most importantly points to the fact that an initial state of a personality construct is no warrant for the rate of change to the level of the construct through time. That would again indicate why an analytic approach like FCM is required to explore language learners’ PE and CI over time and not in one measurement occasion.

The observed fact that the initial high level of CI or PE is not necessarily accompanied by a steeper increase in these subdomains of grit during the L2 course might be explained by the dysfunction of L2 grit levels for the gritty students (Credé et al., 2016). That is, high levels of grit may become dysfunctional if they lower the opportunities of help-seeking behaviors of learners or if they enhance the chances that an individual learner persists too long to solve a problem which is particularly hard rather than spending their time concentrating on other problems that can be easily solved (see Lucas et al., 2015).

There are also certain variables that can affect learners whose PE and CI are low at the beginning of an L2 course more than peers. One key variable involved is the mediating role of the teacher which can be hypothesized to cause a faster growth of efforts and interest in learners who are not as effortful and interested as peers at the beginning of the course. The role of teacher support for autonomy and learners’ engagement was also emphasized by (Yoon et al., 2020) as a mediating factor between learners’ grit and academic success. Thus, it can be postulated that regardless of the level of L2 learners’ PE and CI in the initial session of the course, teacher support and learners’ high level of engagement, aroused by challenging tasks introduced to the course, can intensify learners’ efforts and interest in later sessions of the course.

The learner-centered learning and teaching also account for the intensification of learners’ PE and CI due to the inherent respect for self-directed learning (Yoon et al., 2020). The L2 courses visited for the purpose of this research adopted a communicative language teaching (CLT) approach, which is largely learned-centered (Larsen-Freeman, 2000). Thus, the self-directed learning at the core of this approach might have helped learners whose PE and CI were low at the beginning of the course to increase during the course faster than those whose PE and CI were at a higher level in the first session. Further qualitative research can help to substantiate these possible mediating factors.

The other variable which can help to explain the negative covariance of the intercept and slope of L2 learners’ PE and CI in the FCM of this study is mindset, which in the literature, has been often linked to learners’ grit and has also shown to be causally related to it (Frydenberg, 2017). Khajavi et al. (2020) depicted L2 learners’ perception of their L2 learning ability associated with their efforts in learning the L2. This association has been verified by some studies which mindset as individuals’ perceptions of their language learning ability (e.g., Mercer and Ryan, 2009; Ryan and Mercer, 2012; Lou and Noels, 2017). Thus, we can conjecture that the observed different rate of growth in L2 learners’ PE and CI between those at a low initial level of the subdomains and those at a high initial level of PE and CI in the EFL course could be due to their language mindsets.

In the same line of discussion, we can argue that the steeper increase of PE and CI in learners whose initial level of grit subdomains was lower can be due to the correlation between these dimensions of grit and situational-specific emotional states (Datu and Fong, 2018). Considering the recent research on the dynamics of emotions in SLA (e.g., Gkonou et al., 2017; Boudreau et al., 2018), the link between positive emotions and grit (Datu et al., 2018), and the contribution of L2 teachers to learners’ moments of enjoyment (Dewaele et al., 2018), it can be hypothesized that L2 learners’ emergence of positive emotions influenced by teachers in different conditions of an L2 course, can pave the way for change in efforts and interest of less gritty learners during the course.

Thirdly, we analyzed the *between*-subdomain covariances as the basis of our PPM. The results showed that the covariance of the intercepts and slopes of the two subdomains (i.e., PE and CI) was positive and statistically significant. This is justified as PE and CI are the subdomains if a single global factor (i.e., grit) and the significant positive correlation of these two subdomains attest to the fact that these two, hand in hand, comprise a single underlying latent variable. Interestingly, this association is moderate. If it showed to be high, that would imply the two subdomains could be integrated as one. If it were low, that would question the internal validity of the construct (L2 grit). Yet, the moderate correlation substantiates the construct validity of the latent variable and its two components which are adequately independent and at the same time positively associated.

A further point concerning this third finding was that the covariance of the slopes was higher than that of the intercepts. This finding shows that regardless of the differences in the initial level of PE and CI, the rate of growth in the two subdomains has enjoyed a stronger correlation. In other words, learners at a more or less level of PE and CI to begin with managed to grow efforts and interest to a similar rate. Considering the most influential variables on grit, including teacher’s motivational role, introduction of challenging tasks, and use of a more learner-centered teaching, it seems that the two subdomains of L2 grit are affected (positively) to a similar degree. That is to say that teacher’s mediating role has managed to raise language learners’ interest and at the same time and to a similar degree motivate them to invest efforts in the language learning process. Evidently, in the dynamic context of an L2 class, many relevant variables can work in concert to motivate learners to persist in passion for learning the language and put more efforts in the tasks. Further research can help to reveal more about the factors affecting and affected by L2 learners’ grit in the dynamic context of an L2 class.

Fourthly, we analyzed the contribution of the global factor (L2 grit) to the variance of each of the primary intercept and slope factors. The analysis results showed that the variance of the intercept and slope of each subdomain (PE and CI) was to a large extent explained by the underlying global factor. This would act as a CFA of the construct and shows that the subdomains and their co-development are all subsumed under a single latent factor which is L2 grit.

Overall, the FCM procedure showed to be a privileged and comprehensive analytical approach that enabled us to explore the co-development of L2 learners’ PE and CI in the dynamicity of a language class. It proved effective in revealing inter-individual variation and the results of testing the effect of the global latent variable on the initial state and intercept of the subdomains as they naturally developed in the temporal phases of a language course. Additional qualitative phases can reveal more about the reality of classroom learning and reveal more of the intertwined network of learner-related and contextual factors.

## Conclusion

Considering the dearth of research on grit in general and L2 grit in particular, the dynamic nature of SLA and the concomitant methodological turn in related research (Gass and Plonsky, 2020), it is essential to explore L2 learners’ grit longitudinally to capture a more realistic, developmental picture of the construct as it evolves in the live classroom setting. The measurement is expected to reflect this dynamic turn. The statistical model employed is expected to represent the co-development of the subdomains of the construct and be sensitive to the initial level as well as the growth of change in the subdomains. It is expected to take into account inter-individual variation and the contribution of the global latent variable to the variation in the growth of primary subdomains. Conventional statistical methods fail to realize all these and, thus, only provide a static view of the target trait which is far from its developmental nature within the ecology of classroom. Conventional methods, by taking mean scores of groups as representative of individual performance or trait, ignore inter-individual variation which naturally characterizes the development of human traits or behaviors. The present study revealed these inter-individual differences and drew attention to the need for further qualitative phases or case studies to delve into the matter. The present study warned against the consequences of conventional statistical procedures to explore multidimensional human traits including grit. It emphasized the essentiality of avoiding a mere integrative and holistic approach to the trait and instead the need for tracing the trajectory of changes in each subdomain and check for the covariances. The present study drew attention to the hazards of sufficing to single-shot research designs and the static unrealistic image of the trait they provide which can lead to inconclusive decision making about individual language learners.

The FCM used in the present research can be employed as effectively to explore other human traits in positive psychology including enjoyment, growth mindset, and wellbeing. In the dynamicity of a language classroom, marked by the interaction of many individual (teacher/learner) and contextual factors, the FCM can be efficiently used to trace the growth of the construct of interest in the temporal stages of the course nested in the dynamic reality of classroom learning. Even among innovative statistical procedures within the SEM framework especially LGM, the FCM has the privilege of going beyond the exploration of univariate models and instead aims to trace the co-development of the subdomains of a construct. It also benefits from the analysis of second-order variables (i.e., the intercept and slope of the underlying latent variable). It best suits the measurement of multidimensional human constructs from a CDST approach and can, thus, significantly enrich the existing body of research in SLA domain, which has long shown interest in language learners’ personality constructs, such as motivation, attitude, mindset, and grit.

## Data Availability Statement

The raw data supporting the conclusions of this article will be made available by the authors, without undue reservation.

## Ethics Statement

The studies involving human participants were reviewed and approved by the Changchun Institute of Technology. The patients/participants provided their written informed consent to participate in this study.

## Author Contributions

RW, MS, and TT participated in the data collection of the study. RW, MS, and TT read and approved final manuscript. All authors contributed to the article and approved the submitted version.

## Conflict of Interest

The authors declare that the research was conducted in the absence of any commercial or financial relationships that could be construed as a potential conflict of interest.

## Publisher’s Note

All claims expressed in this article are solely those of the authors and do not necessarily represent those of their affiliated organizations, or those of the publisher, the editors and the reviewers. Any product that may be evaluated in this article, or claim that may be made by its manufacturer, is not guaranteed or endorsed by the publisher.
